# Polyurethane composite behavior influenced by the characteristics of the employed bentonite filler

**DOI:** 10.1038/s41598-025-18822-0

**Published:** 2025-09-29

**Authors:** Alpár Ferencz Hatvani-Nagy, Ferenc Kristály, Béla Viskolcz, Béla Fiser

**Affiliations:** 1 Institute of Chemistry, University of Miskolc, Miskolc-Egyetemváros, 3515 Hungary; 2 Higher Education and Industrial Cooperation Centre, University of Miskolc, Miskolc-Egyetemváros, 3515 Hungary; 3 Institute of Mineralogy and Geology, University of Miskolc, Miskolc, 3515 Hungary; 4 Ferenc Rakoczi II Transcarpathian Hungarian College of Higher Education, Beregszász, Transcarpathia 90200 Ukraine; 5 Department of Physical Chemistry, Faculty of Chemistry, University of Lodz, Lodz, Poland

**Keywords:** Polyurethane composite, Rheology, Clay minerals, Foam analysis, Compatibility, Foam stability, Mechanical properties, Composites, Materials chemistry, Polymer chemistry

## Abstract

Bentonite clays are widely used in polymer composites due to their ability to enhance mechanical and thermal properties. This study investigates the impact of various bentonite types on the rheology of polyurethane (PU) foam formulations and the stability of the resulting foams. Seven different bentonite samples, including both raw and activated forms, were characterized by using X-ray diffraction (XRD) and incorporated into polyurethane premixes. Rheological analysis revealed a significant correlation between bentonite content and viscosity hysteresis, which influenced foam stability. Results showed that certain bentonite types, such as natrium-activated bentonite from Hungary (B3 and B5), improved foam integrity by maintaining low hysteresis values, while others, such as calcium Romanian bentonite (B1), or Na-Ca-bentonite from Slovakia (B2) and standard Wyoming Na-bentonite from USA (B6), led to foam collapse at higher concentrations. Additionally, compressive strength tests demonstrated that small amounts of bentonite enhanced with 250% or more the mechanical properties of the foam. In the case of natrium-activated bentonite from Hungary (B3), catalytic effect is also revealed, decreasing the reaction time with 20%. This research provides insights into the role of bentonites as fillers in polyurethane composites, identifying key factors that determine their compatibility and usability.

**Supplementary Information:**

The online version contains supplementary material available at 10.1038/s41598-025-18822-0.

## Introduction

Polyurethane is one of the most widely used polymers in modern materials science^1^ offers cost-effective solutions for many industries. Foams represent a significant segment of polyurethanes^2^. Two main components are used as raw materials, a polyether or polyester based polyol and an isocyanate with MDI (methylene diphenyl diisocyanate) or TDI (toluene diisocyanate) base structure^3^. The raw materials are rarely used in a pure form, usually some isomer blend or prepolymer already polymerised to some extent. In addition to the two monomers, a wide range of additives are also used. The most important are foaming agents, catalysts, cellulases, stabilizers and fillers. In terms of foaming, a distinction is made between chemical and physical foaming agents. Chemical foaming occurs due to the expansion of a gas released during the reaction, while physical foaming is initiated by the effect of a readily evaporating liquid being converted into a gaseous state. In the past, hydrocarbons containing chlorofreon^4^ were used as foaming agents, but changes in environmental regulations have led to the use of water, which, when added to the polyol mixture, reacts with the isocyanate group at the moment of mixing, thus ensuring the release of carbon dioxide in the reaction mixture. In addition to the addition of foaming agents to the reaction mixture, mechanical foaming is also used, where compressed air is used to form the cell structure^5^.

Thus, the production of polyurethane foam is the result of the equilibrium of two competitive reactions. The first is the gelation process, where polymer chains are formed from polyol and isocyanate molecules, which form the walls of the foam cells. The other is the water-isocyanate reaction, involving gas evolution, in which hydrolysis of the -NCO groups generate carbon dioxide and closes the chain by forming an amine group. The resulting carbon dioxide inflates the polymer and forms alveoli, the aggregation of which generates foam.

During production, additives are mixed into the polyol. In addition to the raw materials involved in the reaction, surfactants are one of the most important additives^6^. They can be polar or apolar and can be emulsifiers, foam stabilisers or cell structure regulators depending on their role in the synthesis. The primary function of the emulsifier is to emulsify the water-organic phase by reducing the surface tension. Usually, these materials also have a stabilising effect. The foam stabilisers form a monolayer on the phase surface, which provides elasticity^7^. The resulting surface elasticity maintains the cell structure until the foam solidifies. The last phase of foam formation is the most critical, as it is here that the amount of gas released into the disperse system reaches its maximum. At this point the cells can rupture, so the role of these additives is critical in this phase. The activity of the surfactant affects the expandability of the molecular layer The higher the activity, the more the cell can grow or the less the amount of stabiliser needed. Polysiloxanes or their polyether siloxanes with different chain structures are used for this role^8^. The use of foam stabilisers determines whether the polyurethane will form an open or closed cell structure at the end of the reaction and affects the rheological properties of the PU^9^. The third group of tensides are cell structure regulators. They act in the opposite way to foam stabilisers, causing the cells to partially collapse and produce a spongy irregular structure. They are used in the production of cold foams. Usually, poly methylsiloxanes or copolymers thereof^10^.

Polyurethane foams do not always meet industrial requirements, and their mechanical properties need to be improved. This can be done by physical and/or chemical means, using fillers. Fillers increase the stiffness, heat and chemical resistance, compressive and tensile strength of PU composites and in most cases reduce the cost of production^11^. They can be of organic origin, such as melamine, vegetable powders or inorganic, grinded minerals, glass beads, etc.

In terms of form, we talk specifically about fillers and scaffolds but there is no clear distinction between the two categories. Fillers are usually applied in the form of powders, skeletons in the form of fibers. If such a filler leads to a significant improvement of a property, it is called a polymer-based composite^12^. The aim of this research is to look for correlations between the physico-chemical properties of various inorganic fillers and the stability of polyurethane foams, bearing in mind their applicability^10^.

Inorganic components are relatively often used as fillers in polymer matrices mainly to increase the thermal resistance and mechanical strength of composites^13^. Such mineral fillers include silica, diatomite, zeolite, clay minerals and other natural fillers. From these categories, materials for which molecular interactions can occur have been selected to significantly increase the compressive strength and stiffness of foams^13,14^. Clay minerals were selected, in particular bentonites, whose layered structure shows potential compatibility with the polyurethane matrix and increases the range of applications. The first polymer-clay nanocomposites were developed by Toyota researchers in 1993 using Nylon 6, opening a new path in the field of structural materials^15^. The first polyurethane-clay nanocomposite was synthesised by Wang and Pinnavaia in 1998^16^. Among the clay minerals, the role of layered silicates as fillers has been the most studied, but composite fabrication experiments have also been carried out with hectorite and laponite. Bentonite is named after the US town of Fort Benton, near which large quantities of the rock are found. Its main constituent mineral is montmorillonite which is a phyllosilicate with a layered composition of (Na, Ca_0,5_)_0,33_(Al, Mg)_2_Si_4_O_10_(OH)_2_ - nH_2_O, whose basic unit consists of a repeating series of two SiO_4_ tetrahedra and a layer of Al_2_(OH)_3_ and Mg(OH)_2_ octahedra (T-O-T structure). The layers are dominated by negative charge due to isomorphic substitution, balanced by metal ions in the interlayer space. For example, a mineral is called Na-bentonite according to the nature of the dominant metal ion if the majority of the interlayer space is occupied by Na^+^ ions. These compensating charges are attached to the layers of the crystal lattice by ionic bonding and are exchangeable depending on the reaction conditions^17,18^.

Clay fossils are divided into three large families according to the thickness of the layers. They can be 7, 10 or 14 Å, with tetrahedral Si and/or octahedral Al^3+^, Ni^2+^, Mg^2+^, Ca^2+^, Fe^2+^, Fe^3+^, Mn^2+^, Na^+^, K^+^ arrangement. Phyllosilicates such as bentonite occur naturally as microcrystals, hexagonal plates or microfibres (e.g. halloysite, kaolinite, montmorillonite, illite, glauconite, chlorite, various smectites, and vermiculites). The so-called interstitial space between the layers can bind water and, as mentioned above, ions. As a result, the distance between the layers can vary and the mineral is therefore prone to hydration (swelling) or desiccation (contraction).

During the current study, six different types of bentonites in polyol mixtures were examined, the corresponding foams were prepared, and the rheological properties were correlated with the foam properties. The foam samples that retain their integrity were subjected to mechanical tests, monitoring the variation of compressive strength as a function of composition.

## Materials and methods

In our study, six bentonite types and pure montmorillonite were investigated and their applicability in composite formulations were compared (Table 1).

**Table 1:** Different bentonite types used in polyurethane composite formulation.

Number	Bentonite type	Code
1	Raw bentonite Satu Mare country, Romania (Bentoflux SA)	B1
2	Raw bentonite Mutnik Hnusta country, Slovakia (Gemerská Nerudná Spoločnosť A.S.)	B2
3	Activated bentonite Mád, Hungary (Geoprodukt)	B3
4	Raw bentonite Rákóczibánya, Hungary (Salakfeldolgozó Kft.)	B4
5	ON70 activated foundry bentonite Mád, Hungary (Geoprodukt)	B5
6	Wyoming standard raw bentonite, Wyoming USA (Wyo-Ben)	B6
7	K10 Montmorillonite, Sigma Aldrich (Süd Chemie)	MMT

During production, the filler is the first to come into contact with the polyol. This water-soluble phase contains the catalyst, foaming agents and water. The bentonite was mixed to form a premix, which subsequently reacted with isocyanate. During the design phase, an experimental matrix was set up to analyse the effect of the minerals on the flow properties of the premixes. The premixes were subjected to rheological analysis, investigating the variation of stresses in the fluid as a function of shear rate at more than 1700 points.

### Material preparation

During the preparatory phase of the experiments, we placed great emphasis on consistent material quality, which is the cornerstone of reproducibility. All fillers were subjected to the same treatment, and more than the foreseeable amount was produced. The samples were homogenised to ensure the same composition for all experiments.

The bentonites were first pulverized and then left uncovered on watch glass for a week at 50% humidity in air at 22 °C to allow equilibrium humidity to set and to minimize damage to the crystal structure. After stabilisation of the mass, the bentonite was ground in a porcelain mortar and sieved through a 63 µ sieve. The passing fraction was hermetically packed, and the remaining fraction was subjected to repeated grinding and sieving to ensure homogeneity of the bentonite particle size. This fraction is generally used in industry, like a particle size that conferred optimum properties for bentonite powder. To prepare polyurethane foams the Ongronat TR4040 (BorsodChem Zrt, Kazincbarcika, Hungary) with FFP-303 was mixed. The latter is a preformulated polyol mixture with the following components: Wanol F3160 (polyether polyol, Wanhua, China), Tegostab B4113 surfactant (polysiloxane, Evonik, Germany), Tegoamin DEOA 85 catalyst (Evonik, Germany), Dabco 33LV gelling catalyst (Evonik, Germany) (1,4-Diazabicyclo[2.2.2]octane) and Jeffcat ZF22 catalyst (Hunstman, Texas, USA).

### Exploring the structure of bentonites

One of the most important techniques used to identify and/or analyse clays is X-ray diffraction analysis. There are several X-ray diffraction methods for the analysis of crystalline and amorphous solids, liquids and gases. For crystalline materials, the most common methods are single crystal and powder diffraction analysis. In the XRD technique, a key step is sample preparation^19^.

The sample is suspended in water, then settled for a period of time and only the fraction below 2 µm is recovered by decantation of the upper liquid phase, which is considered to be the fraction containing the ‘pure’ clay phase. This fraction in solution is then placed on a glass plate and dried. During drying, the lamellar clay particles are oriented according to their crystallographic (001) plane. When this plate is subjected to XRD analysis, only the 001 baselines are obtained (i.e. 001, 002, etc.). These are characteristics of clay particles^20^.

X-ray diffraction measurements and polycrystalline techniques were used to characterise the bentonites. The samples were dried in a drying oven at 105 °C to constant mass in a mortar and then sieved, the fraction below 63 µ was refined in an agate mortar. The average particle size was 20–45 µ. The ground refined clay was placed in Eppendorf tubes, hermetically sealed, and then applied to a polished glass slide in a stainless-steel sample holder, subjected to rotational diffractometric analysis at a wavelength of 1.54060 nm. For analysis we used a Bruker D8 Discover XRD diffractometer equipped with ATLAS goniometer. The center hub includes a high precision bayonet stage mounting mechanism to ensure the sample stage is positioned exactly in the center of the goniometer.

### Composite preparation

The selected bentonite minerals were mixed with FFP-303 preformulated polyol and allowed to swell for 24 h in airtight containers. The added bentonite content was calculated for the final weight of the composites which was prepared by mixing the FFP-303/bentonite with 22.5 g MDI based isocyanate TR-4040 (Table 2). The final composite would contain exactly 1, 3, 5, 10, 15, and 20% of minerals.

**Table 2:** Applied bentonite quantities during the preparation of the composites.

Premix bentonite content (%)	Monomers (g)	Bentonite quantity (g)
1	37.5 g polyol + 22.5 g isocyanate	0.606
3		1.855
5		3.157
10		6.665
15		10.588
20		15.000

The PU foams were prepared by stoichiometric dosing with NCO index 1.0, adding 22.5 g isocyanate to 37.5 g polyol. Tegostab B4113 emulsifier was used to stabilize the foam, while a commercially available chemical mixture DABCO 33LV containing 33% triethylene diamine and 67% dipropylene glycol was employed as catalyst. Rokopol was used as a crosslinker, DEOA 85 diethanolamine solution as a catalyst, and bis-(2-dimethylaminoethyl) ether molecule commercially branded as Jeffcat ZF 22 low VOC emission catalyst to enhance the catalytic effect.

Sartorius analytical balance was used to measure the raw materials. The foam growth rate was determined with a FOAMAT 285^21^. The foams were prepared in 750 ml paper cups. The amount of bentonite varied, while the amount of FFP in the premix and the amount of MDI prepolymer isocyanate TR 4040 added was constant for all composites. After mixing the components, the mixture was intensively stirred for 15 s (1200 rpm) and then, foam formation was followed for 240 s. The FOAMAT instrument measures the instantaneous reaction rate, foam density and foam height.

### Rheological characterization of premixes

Rheological studies are important in polyurethane synthesis because the two antagonistic processes, gelation and gas formation, are only possible under certain rheological conditions^22^. The flow parameters of the original polyol-isocyanate system change significantly with the addition of bentonite. On the one hand, the mere physical presence of the clay mineral as a solid increase the viscosity of the medium^23^. On the other hand, the rheological interactions are crucially influenced by molecular interactions. In the creation of premixes, bentonite is mixed with polyol, into which isocyanate is introduced. The polyol is not always a neutral medium for bentonite, some bentonites can form inclusion compounds with polyalcohol molecules, significantly increasing the viscosity and elasticity of the reaction bed^24^. In our case, there is a difference between the ascending and the return branch of the tensiometric curve of the tangential shear rate, which is plotted in a graph based on the Ostwald-de Waele Equation^25^, and which increases and decreases in steps, which is called hysteresis^26^. This phenomenon is characteristic of time-dependent fluids with non-Newtonian behaviour^27^. The area bounded by the two curves, which can be quantified by the concept of hysteresis, characterizes the intrinsic elasticity of the fluid under mechanical stress. A higher value of hysteresis suggests that it takes more time for the molecules of the fluid to relax at the shear stress at the original measuring point^28^. Therefore, the viscoplastic component of the fluid is dominant. A lower hysteresis value, on the other hand, implies a faster back-straightening, where the elastic component is more dominant (Maxwell model)^29^.

The theoretical phenomena observed in premixes are not limited to viscoelastic processes. The experiments have shed light on an interesting rheological property of premixes. These fluids have a flow threshold. If the graphs of the Ostwald-de Waele equation are described by a linear function, the coefficient of the variable gives the slope of the line, and the free term gives the yield point^30^. If the shear rate is set equal to zero, the value of the stress corresponds to the value of the free term. For viscoplastic liquids (such as premixes), the minimum shear stress at which the liquid starts to flow is called the yield stress. If the shear rate is zero, the liquid does not move, but as soon as this value starts to increase, i.e. the minimum movement starts, the starting point of the tension curve can only be at the vertical axis intercept, i.e. the value of the free member corresponds to the flow threshold (Burgers or Herschel - Bulkley model). Polyurethane composite foams were prepared from the premixes and the relationships between foam stability and the aforementioned rheological properties were investigated, defining the basic requirements for the integrity of the foam structure. The formulated polyol-bentonite premixes were prepared for all bentonite types (B1, B2, B3, B4, B5, B6, MMT), yielding a total of 42 mixtures, each of which was rheologically tested with a minimum of 40 measurement points, depending on the measurement limit. In addition, the rheological behaviour of pure FFP was investigated at 48 measurement points. A Searle type rotary rheoviscosimeter^31^ with a thermostatic jacket connected to an aqueous ultrathermostat was used to condition the samples to 20 °C. In all cases, the mixtures used were homogeneous and free of sedimentation. The used equipment was a Rheotest-2 rheoviscosymeter by MLW Medingen Prüfgerate Germany. The shear rate varied in increasing and decreasing steps, measuring the angle of regression over the entire measuring range. From these values, shear stress and dynamic viscosity were calculated. The hysteresis of the stress and viscosity in each premix was determined by measuring the area between the ascending and descending branches of the tension curve. The rate of change of stress and yield threshold were determined by mathematical modelling of the tension lines.

### Measurement of compressive strength

For mechanical measurements, cylinders with a diameter of 30 mm were cut from the composite samples with 50 mm height. The circular bases of the cylinders were cut parallel and perpendicular to the slate generator, removing at the same time the surface skin layer, which introduces measurement uncertainty into the system. To determine the compressive strength, a Zwick-Roell brand mechanical testing machine^32^ was employed which measures these parameters with high accuracy. In case of the analysis, the ASTM D3574 testing method was used to determine the mechanical properties of the samples^33^.

## Results and discussion

**Fig. 1:**
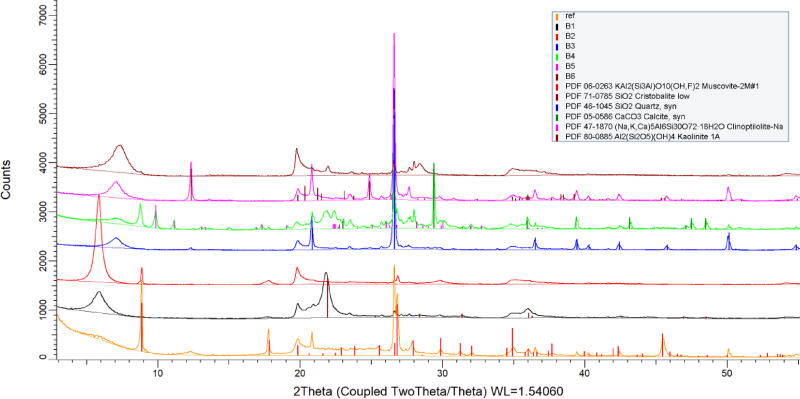
XRD patterns of the studied bentonite types (B1-B6, MMT).

The aim of the research is to find correlations between foam stability and filler properties and to investigate the rheological behaviour of bentonites and their modifying effect on foam stability. At first because the research is focused on fillers, we characterized the bentonites and synthetized the foams and by premix analysis we were looking for answers to explain the phenomena we were experiencing. The bentonites conditioned as described in the sample preparation were subjected to XRD analysis. Basically, two characteristic curve types were found, regarding the montmorillonite type: the first being the 14Å Na-montmorillonite (Wyoming type, B1 and B2), the other the 12Å Ca-montmorillonite (B3, B4, B5, B6) bearing bentonites shown in Fig. 1.

Beyond montmorillonite we identified muscovite, silicon dioxide as cristobalite and quartz, calcite and albite. The highest proportion is 14 Å montmorillonite between 5–9° 2Theta with a symmetrical and sharp peak for the Na-type. The presence of albite is detected as an accessory mineral. This mineralogical composition is general, with the given variations.

There are two other bentonites, B3 and B5 with similar XRD pattern, from nearby deposits that have very different parameters from the above composition (Fig. 1). The pattern illustrates the presence of a 400-intensity montmorillonite peak at grid spacing 12 Å and several lower intensity albitic, kaolinitic and orthoclase peaks, which are of primary volcanic origin slowly transformed into montmorillonite from the granitic, dioritic rocks. What is unique in these rocks, however, is the presence of high quantity of microcrystalline quartz with high intensity proportion, indicating its significant amount in the material. These needle-like crystals wedged between the montmorillonite crystals change the traditional behavior of the clay rock.

**Fig. 2:**
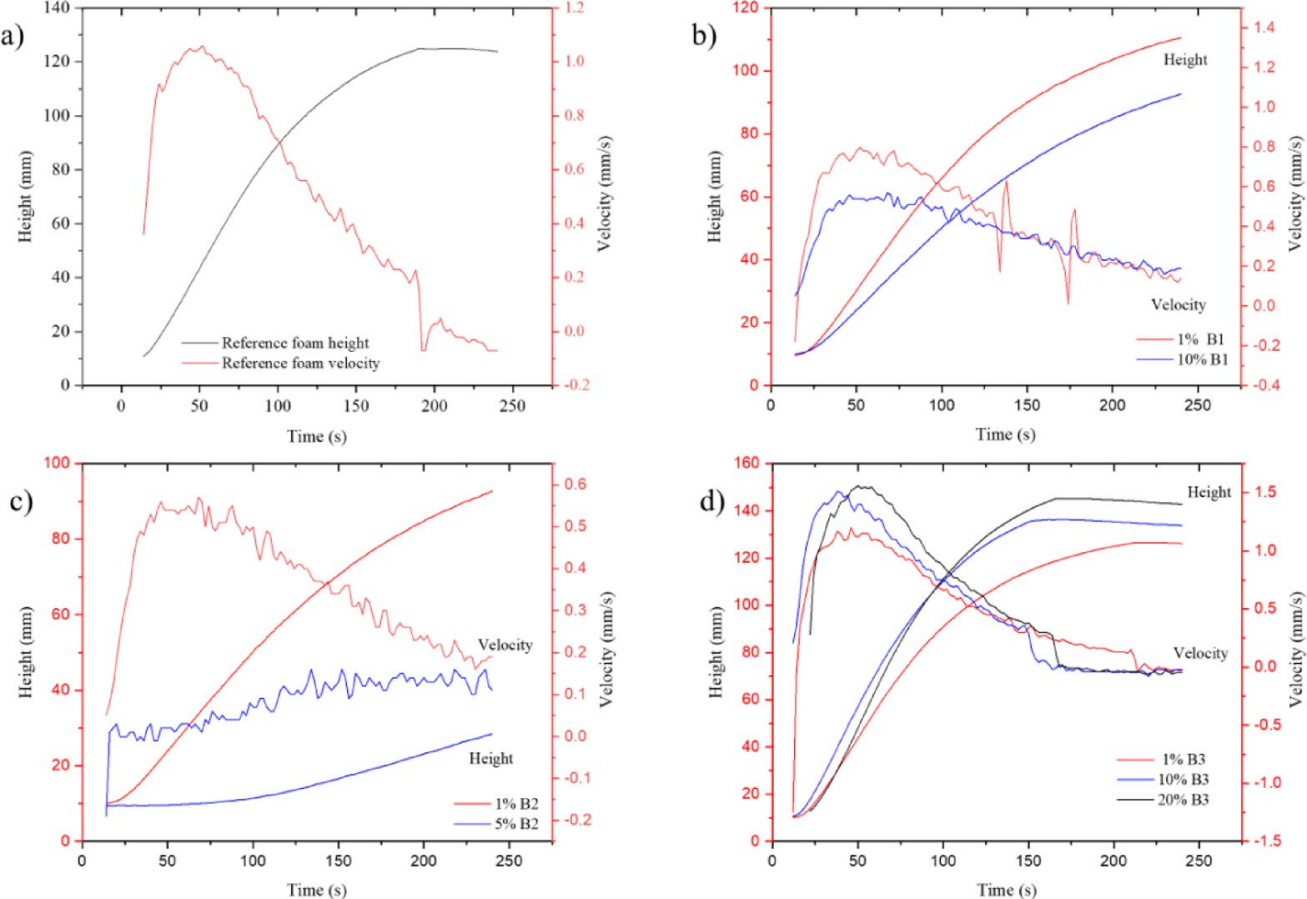
Formation of polyurethane foams and composites containing bentonites measured with the FOAMAT.

Premixes were prepared from the previously conditioned and XRD characterized minerals, from which composite foams were synthesized after 24 h swelling with the above-mentioned filler compositions of 1, 3, 5, 10, 15, and 20%. Among the FOAMAT curves describing the foam formation process, the development dynamics of the reference foam without filler were first investigated (Fig. 2a).

In the graphs, the red curve shows the instantaneous reaction rate, and the black curve shows an increase in foam height. For standard PU foam, the maximum reaction speed is 1.03 mm/s. After reaching the maximum around 50 s the reaction slows down and after 240 s of tracking time the foam reached a height of 123.9 mm.

The first filler tested was bentonite B1. Added in a small proportion to the original formulation, it slightly reduces the foam height and slows down the reaction (Fig. 2b). At 1–3%, it does not impair foam formation but increases the compressive strength of the foam. If the amount of B1 bentonite exceeds 5%, the reaction slows down considerably, we should observe that the blue curve is undre the red. The phenomena resembling the terminal phase of the reference graph, the foam growth phase increases to 10 min and, after an initial apparent stability, the foam collapses within 24 h. The collapse ends with the break-up of the cell structure and the formation of irregular agglomerates. It is observed that the reaction rate curve shows oscillations even at a filler content of 1%, caused by foam the surface instability, bulb formation.

A similar phenomenon is observed for B2 bentonite (Fig. 2c), where the above phenomenon occurs even earlier. The 3% sample already shows practically a slowed down reaction and although the foam increases to a lesser extent compared to the reference, the collapse occurs within one day.

The experiments with B4, B6, MMT fillers gave similar results to the previous ones, but different foam growth curves were recorded in the experiments with B3 activated bentonite.

**Fig. 3:**
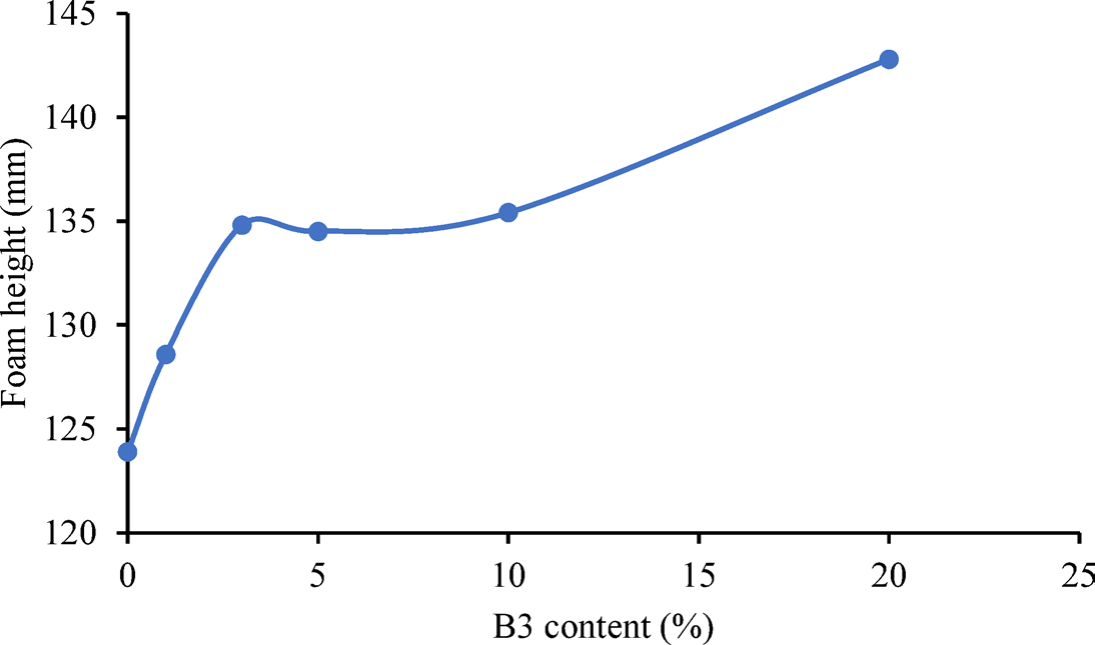
Foam height vs. B3 bentonite content.

In case of experiments with B3 bentonite (Fig. 2d), the effect of different bentonite percentages on the composite foam was observed. The reaction rate during the foaming process is evaluated by monitoring the temporal variation of foam rise. A higher foam rise rate corresponds to a faster reaction. To a lesser extent, we observed a similar effect with B5 bentonite. For B3 bentonite, the reaction rate increases with increasing bentonite content. The maximum is 1.5 mm/s which is significantly higher in comparison to the reference foam. The black curve exhibits the highest rate with highest B3 content, followed by the blue and then the red curve with decreasing rates. The B3 accelerated the reaction, with the produced foams reaching their final height in 144–152 s instead of 180 s on average and increasing in size compared to the initial rise. The reaction time decreased by 20%. The final foam height was plotted against the B3 content and the graph clearly shows that the rise of the B3 composite foam is significantly greater (15% increase for the 20% B3 composite) compared to the 123.9 mm height (0% B3 content) of the reference foam (Fig. 3). No collapse occurred at higher bentonite contents, and the additive has a significant reaction accelerating effect, and the cell structure is retained. Because the mass was maintained at the same value, and the volume of the foam increased with height, the added bentonite decreases the density and generates a more flexible easy foam with better productivity. The catalytic effect of bentonite can be due to the microcrystalline quartz revealed by XRD analysis, which accelerates polymerization by increasing the number of reaction sites. Regarding the catalytic effect, it is primarily based on physical mechanisms. The crystal structure of B3 uniquely contains a significant amount of microcrystalline quartz (Fig. 1). Although quartz is not a catalyst in the chemical sense—since it does not intervene directly in the reaction mechanism—the quartz particles can act as nucleation centers. The reactants (isocyanate and polyol) are adsorbed onto the SiO₂ surface, which leads to locally increased concentrations. Along these surfaces, the reaction proceeds faster due to the reduced intermolecular distance between reactant molecules. This effect may be particularly important during the initial stages of foam formation, where the reaction is exothermic, and quartz can additionally stabilize the reaction zone by acting as a thermal conductor. Since quartz is present in large amounts and in a singular phase, this phenomenon manifests as an indirect acceleration of the reaction^34^.

However, rheological processes have a decisive influence on the retention of foam structure. Based on the FOAMAT experiments the integrity of the final composite products has been compared (Table 3).

**Table 3:** Composite integrity table where “+” indicates retained structure and “-” indicates collapse.

Bentonite	1%	3%	5%	10%	15%	20%
B1	+	+	+	-	-	-
B2	+	-	-	-	-	-
B3	+	+	+	+	+	+
B4	+	+	+	-	-	-
B5	+	+	+	+	+	+
B6	+	+	+	-	-	-
MMT	+	-	-	-	-	-

**Fig. 4:**
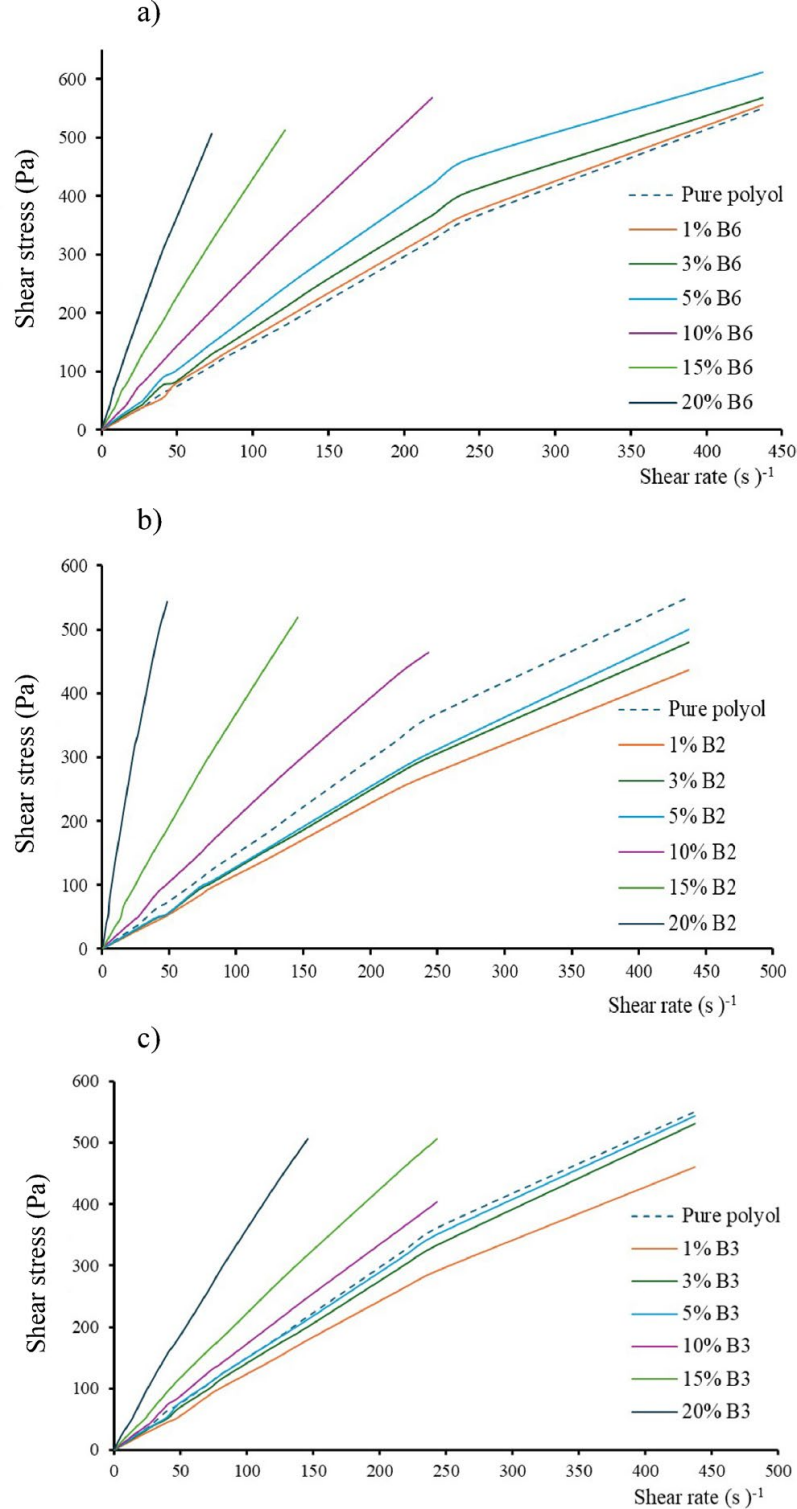
Shear stress–shear rate curves as a function of bentonite content for (**a**) B6, (**b**) B2, and (**c**) B3.

Characterization of the premixes containing catalyst, emulsifiers and fillers after 24 h of resting with a coaxial cylindrical rotational rheometer showed as a general characteristic that in all cases after a certain amount of added bentonite the viscosity of the system increased. The effect of B6 bentonite on premixes was also investigated (Fig. 4a). The addition of bentonite increases viscosity and stress in the fluid as expected. The increasing proportion results in steeper and steeper curves. The classical explanation for this effect is the increase in the number of friction points between polymer chains, which generates increasing stress in the system.

However, the change in rheological behaviour is less clear for other bentonites (Fig. 4b and c). Only premixes above 10% of bentonite content show an increase in internal stress compared to the reference in case of B2 and B3 bentonites. 1% bentonite significantly reduces internal stress, ultimately intermolecular slip, but this phenomenon is not consistent with the foam integrity table, and it is not possible to determine why foam collapse occurs.

**Fig. 5:**
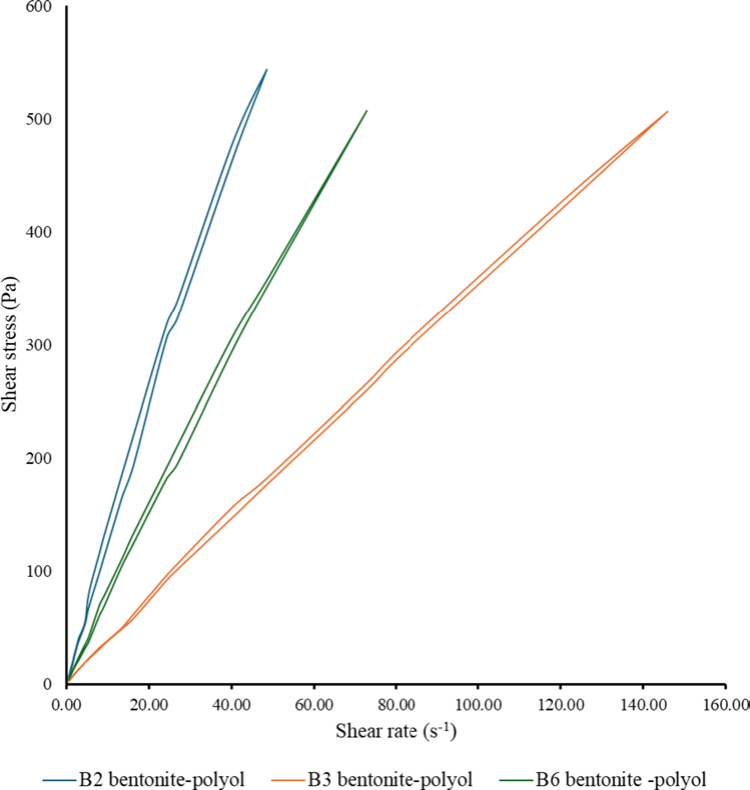
Shear stress hysteresis for B2, B3 and B6 bentonite-polyol premixes.

A deeper study of the phenomena becomes possible if we look at the values not only on the increasing but also on the decreasing side of the shear stress variation as we should observe in Fig. 5. Compared to Newtonian linear classical rheology, the premixes tend towards the higher tension range, the line has a slight curvature, suggesting pseudoplastic behaviour. If we add to this the difference in the time factor between the ascending and the recurrent branches, we arrive at the time-dependent thixotropic behaviour that in fact characterizes the premixes. The degree of thixotropy can be described by the area between the two curves, defined as hysteresis, measured in Pa.s^− 1^ (Table 4).

**Table 4:** Hysteresis values for bentonite-polyol premixes.

Filler %	0	1	3	5	10	15	20	Bentonite
Shear stress hysteresis (Pa.s^− 1^)	1.810	1.458	2.331	6.619	6.194	2.949	7.370	B1
Viscosity hysteresis (Pa)	0.108	0.110	0.116	0.138	0.230	0.264	0.452	
Shear stress hysteresis (Pa.s^− 1^)	1.810	0.938	5.378	4.654	7.873	4.554	9.857	B2
Viscosity hysteresis (Pa)	0.108	0.109	0.156	0.170	0.184	0.231	1.129	
Shear stress hysteresis (Pa.s^− 1^)	1.810	1.298	1.497	0.938	0.709	2.392	2.490	B3
Viscosity hysteresis (Pa)	0.108	0.078	0.051	0.025	0.030	0.110	0.144	
Shear stress hysteresis (Pa.s^− 1^)	1.810	0.667	1.525	2.697	7.073	6.000	6.226	B4
Viscosity hysteresis (Pa)	0.108	0.059	0.096	0.088	0.193	0.376	0.371	
Shear stress hysteresis (Pa.s^− 1^)	1.810	1.566	0.907	0.667	1.293	1.739	1.769	B5
Viscosity hysteresis (Pa)	0.108	0.084	0.050	0.069	0.020	0.095	0.095	
Shear stress hysteresis (Pa.s^− 1^)	1.810	0.250	0.489	3.376	3.675	2.133	5.237	B6
Viscosity hysteresis (Pa)	0.108	0.002	0.029	0.089	0.155	0.247	0.663	
Shear stress hysteresis (Pa.s^− 1^)	1.810	1.762	3.915	3.150	4.868	6.388	7.883	MMT
Viscosity hysteresis (Pa)	0.108	0.100	0.152	0.166	0.238	0.442	0.702	

Besides the seemingly allegorical changes, two trends can be observed.

**Fig. 6:**
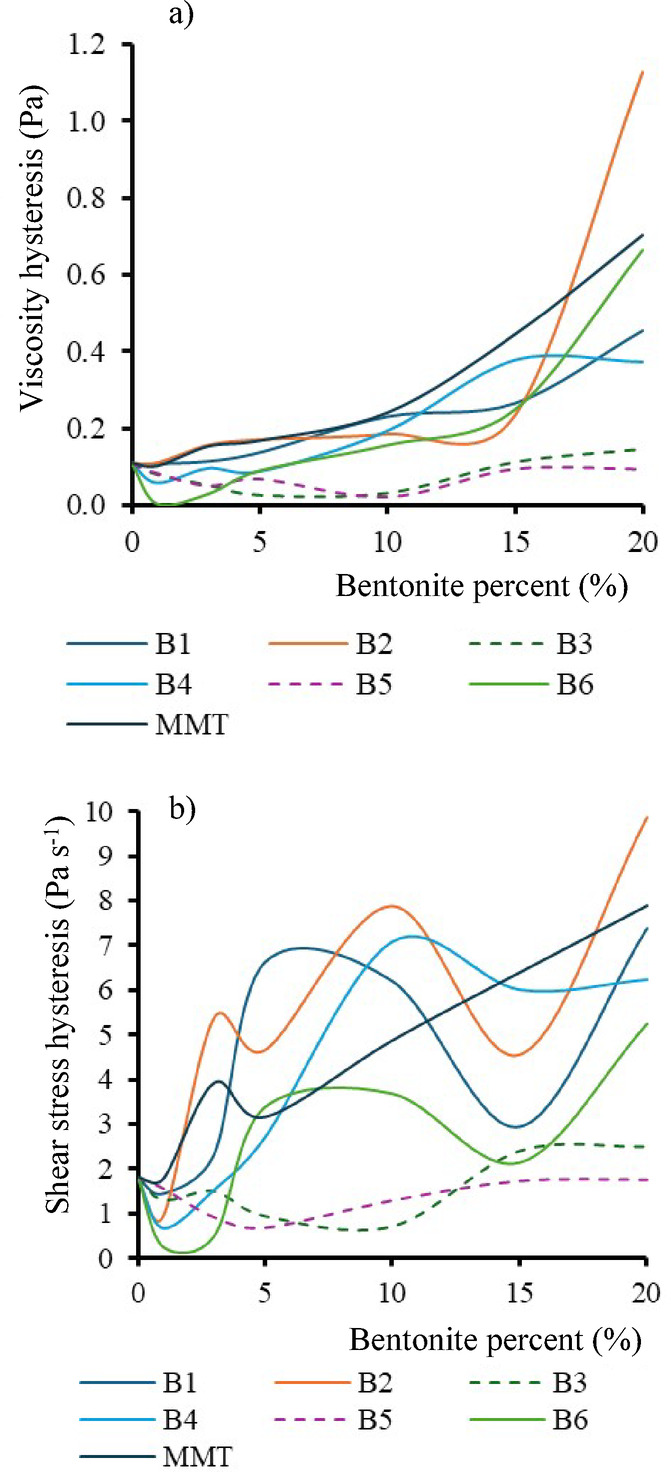
(**a**) Viscosity hysteresis and (**b**) shear stress hysteresis.

The first is that the rate of hysteresis generally increases with the amount of bentonite added. This is an experimentally explainable phenomenon, since the presence of filler particles inhibits the rearrangement of molecules. The actual reason for hysteresis is that during the analysis the molecules do not arrange themselves in the same position on the decreasing shear rate curve at the same rate as on the increasing rate curve. The rotation between the cylinders causes the molecules to become stretched and the rearrangement occurs more slowly, i.e. the process is time dependent. The filler increases the plastic and viscous components of fluid rheology more significantly by reducing the elasticity by stoichiometric inhibition of back-ordering. Second, for foams showing stability, this hysteresis value does not change significantly, compared to the reference premix, despite the increasing amount of fillers added.

By analysing the positions of the curves, Fig. 6 shows that the types of bentonites with the lowest viscosity hysteresis (Fig. 6a) and shear stress hysteresis (Fig. 6b) are B3 and B5, the types with the highest foam stability. The growth of foams is a complex process, which is not continuous, but the formation of the foam structure has growth and relaxation phases which are repeated. The cells that form have increasing stress at the top and decreasing stress at the bottom. In other words, it is not only the specific tensiometry or viscosity value that is decisive in the production of foams, but also the interaction of the plastic, elastic and viscous components of the fluid, which can be best described macroscopically by the concept of hysteresis.

**Fig. 7:**
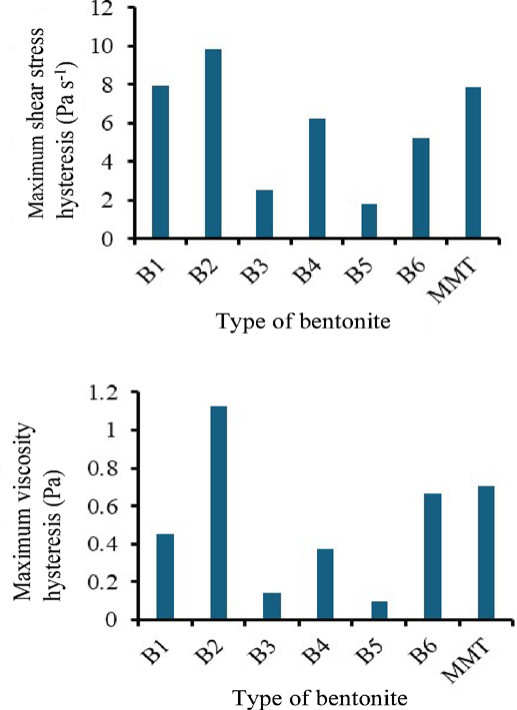
Maximum hysteresis values of the tested premixes with 20% bentonite content, obtained from: (**a**) shear stress vs. shear rate graphs, and (**b**) viscosity vs. shear rate graphs.

In more flexible premixes with lower hysteresis, the bentonite moves with the foam. It does not represent a retention force and does not interact intercalation with the polymers. In foams with a more hysteretic, i.e. viscoplastic component, the cell growth condition is not present, the foam is difficult to grow, the movement of the polymer chains is limited, and the system is typically plastic and inelastic. For viscosity hysteresis, a limit value of 0.15 Pa above which the integrity of the foams is compromised can be established from the data in Table 4. The hysteresis of the original additive-free FFP is 0.1084 Pa. The experimental data suggest that if the increase in viscosity-shear rate between the ascending and the return branch of the viscosity-shear rate rheograms due to the hysteresis filler effect exceeds 38% of the original reference value, it leads to collapse of the polyurethane composite foam. If only the absolute values of the shear stress hysteresis are taken into account, the difference is still striking (Fig. 7). The existence of foam stability is predictable for the smaller hysteresis.

It can be seen that the foams that remained just where the introduced filler increased the viscosity and internal stress hysteresis of the premixes the least.

**Fig. 8:**
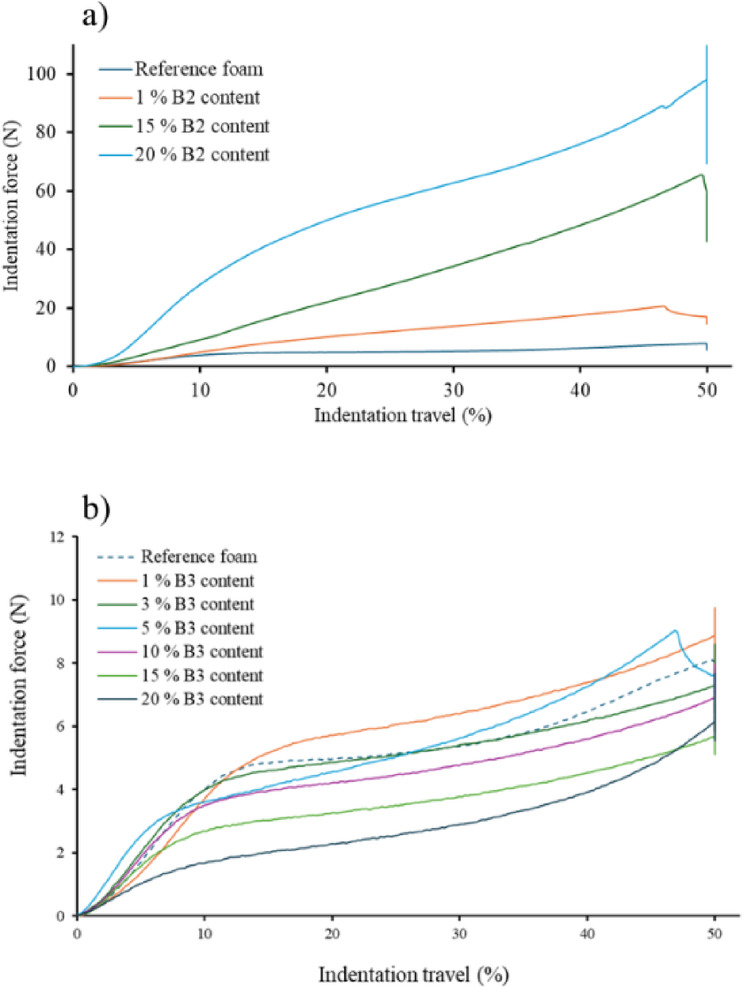
Compressive resistance of composite foams containing: (**a**) B2, and (**b**) B3 bentonites, at different concentrations.

To investigate the mechanical properties of the composites, we compared the compressive strength of foams with different bentonite contents. To illustrate the mechanical properties of the composites, two bentonite types were chosen to illustrate the behaviour of the foams (Fig. 8). Since the same specimen was used for the compressive strength assessment, the composites can be compared.

The B2 is typically a bentonite that causes collapse at as low as 3%. These fillers negatively affect the integrity of the foams in higher quantities, but in small quantities they significantly increase the mechanical properties that are important for users (Fig. 8a). The tested B2 bentonite, when added to 1% of the reference foam, increases the foam strength from 5.87 to 14.65 N. This represents an increase of 250% while maintaining the structure. In the case of composites containing 15% and 20%, although the regular foam structure collapsed, a cellular structure remained which allowed the compressive strength to be measured. The Newtonian values of the indentation force are 42.88 and 69.48, i.e. more than seven and eleven times the compressive strength respectively. These values are of interest not only from a scientific point of view, but also because they allow the synthesis of high-strength flexible polyurethane composites, particularly in the construction industry.

In the case of bentonite B3, no structural damage was observed. In terms of mechanical properties, an increase in compressive strength compared to the original is seen only in the range 1–5%, including a 21% increase from 5.87 to 7.11 N for the 1% composite (Fig. 8b). In this case, therefore, the role of bentonite is not primarily as a strength increasing agent but as a compatibilising agent. The FOAMAT experiments clearly demonstrate that this mineral has a catalytic effect on foam formation and opens up the possibility of producing composites where foam stability is a critical parameter. The proximity of the strength curves demonstrates that bentonite B3 improves or preserves mechanical properties and does not affect cell integrity.

## Conclusions

The study demonstrated the significant impact of different bentonite types on polyurethane composite foam properties, primarily through their influence on rheology and mechanical stability. From an industrial perspective, maintaining foam stability is essential when aiming to produce environmentally friendly foams, and the use of B3 may represent a significant advancement in this regard. However, a collapsed foam structure does not necessarily indicate failure. Rather, it presents an opportunity for the development of high-strength composite materials. The viscosity hysteresis of premixes was identified as a critical parameter influencing foam integrity. Stable foams were associated with lower hysteresis values, while excessive hysteresis led to foam collapse, especially if the hysteresis filler effect exceeds 38% of the original hysteresis reference value. Bentonite types B3 and B5 contributed to stable foam structures even at higher filler concentrations, whereas B1 and B2 caused structural collapse beyond 3–5% content. Some bentonites, such as B3, exhibited catalytic behavior, accelerating polymerization, decreasing the foam density and enhancing foam growth without compromising structural integrity. Small bentonite additions (1–3%) significantly improved compressive strength, with up to a 250% increase observed in some cases. The findings suggest that bentonites can be selectively utilized to fine-tune polyurethane foam properties for applications in construction, insulation, and lightweight composite materials. Future research should focus on the molecular interactions between bentonite minerals and polyurethane precursors to optimize composite formulations further.

## Supplementary Information

Below is the link to the electronic supplementary material.

Supplementary Material 1

## Data Availability

Data is provided within the manuscript or supplementary information files.
