# Supplementary material for: Polyurethane composite behavior influenced by the characteristics of the employed bentonite filler
Source: Sci Rep. 2025 Sep 29;15:33418. doi: 10.1038/s41598-025-18822-0 (PMC12480975; doi:10.1038/s41598-025-18822-0)
Supplement: Supplementary file 1 — Supplementary Material 1 [file 41598_2025_18822_MOESM1_ESM.pdf]

Polyurethane Composite Behavior Influenced by the Characteristics of the Employed Bentonite Filler

Alpár Ferencz Hatvani-Nagy[1,2], Ferenc Kristály[3], Béla Viskolcz[1,2], Béla Fiser[1,4,5,*],

[1] Institute of Chemistry, University of Miskolc, 3515 Miskolc-Egyetemváros, Hungary

[2] Higher Education and Industrial Cooperation Centre, University of Miskolc, 3515 Miskolc-Egyetemváros,Hungary

[3] Institute of Mineralogy and Geology, University of Miskolc, 3515 Miskolc, Hungary

[4] Ferenc Rakoczi II Transcarpathian Hungarian College of Higher Education, 90200 Beregszász, Transcarpathia, Ukraine

[5] Department of Physical Chemistry, Faculty of Chemistry, University of Lodz, Lodz, Poland

bela.fiser@uni-miskolc.hu (B.F.); alpar.hatvani@uni-miskolc.hu (H.N.A.F.); bela.viskolcz@uni-miskolc.hu (B.V.); ferenc.kristaly@uni-miskolc.hu (K.F.)

correspondence: B.F.

Supplementary Information

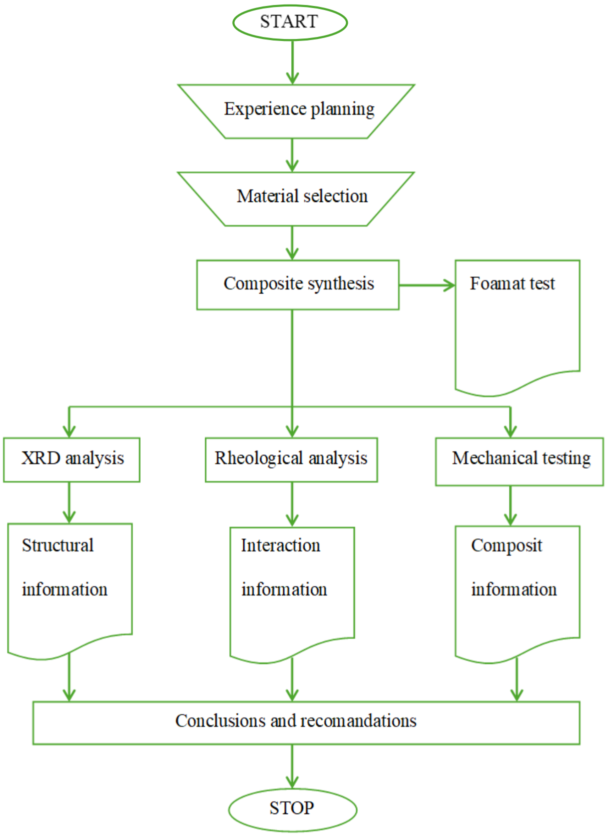

Figure S1 Experimental flow chart

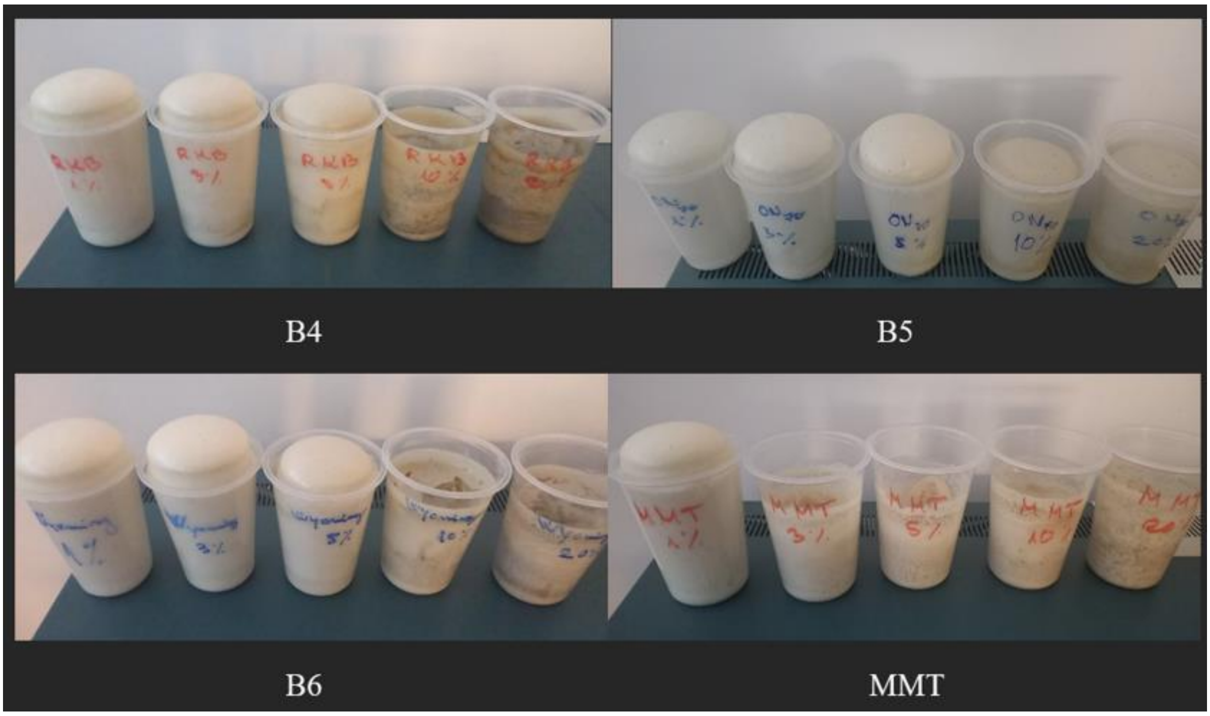

Figure S2 Photos of the prepared composite samples using B4, B5, B6 and MMT minerals.
